# Long-read sequencing for identification of insertion sites in large transposon mutant libraries

**DOI:** 10.1038/s41598-022-07557-x

**Published:** 2022-03-03

**Authors:** Muhammad Yasir, A. Keith Turner, Martin Lott, Steven Rudder, David Baker, Sarah Bastkowski, Andrew J. Page, Mark A. Webber, Ian G. Charles

**Affiliations:** 1 Quadram Institute Bioscience, Rosalind Franklin Road, Norwich, NR4 7UQ UK; 2 Norwich Medical School, Norwich Research Park, Colney Lane, Norwich, NR4 7TJ UK; 3 University of East Anglia, Norwich Research Park, Norwich, NR4 7TJ UK

**Keywords:** Genetics, Genome, Interspersed repetitive sequences, DNA transposable elements

## Abstract

Transposon insertion site sequencing (TIS) is a powerful method for associating genotype to phenotype. However, all TIS methods described to date use short nucleotide sequence reads which cannot uniquely determine the locations of transposon insertions within repeating genomic sequences where the repeat units are longer than the sequence read length. To overcome this limitation, we have developed a TIS method using Oxford Nanopore sequencing technology that generates and uses long nucleotide sequence reads; we have called this method LoRTIS (Long-Read Transposon Insertion-site Sequencing). LoRTIS enabled the unique localisation of transposon insertion sites within long repetitive genetic elements of *E. coli*, such as the transposase genes of insertion sequences and copies of the ~ 5 kb ribosomal RNA operon. We demonstrate that LoRTIS is reproducible, gives comparable results to short-read TIS methods for essential genes, and better resolution around repeat elements. The Oxford Nanopore sequencing device that we used is cost-effective, small and easily portable. Thus, LoRTIS is an efficient means of uniquely identifying transposon insertion sites within long repetitive genetic elements and can be easily transported to, and used in, laboratories that lack access to expensive DNA sequencing facilities.

## Introduction

Transposon insertion site sequencing (TIS) is a robust tool used to identify the genetic loci associated with the phenotype of an organism^1^. The general approach for TIS methods has been developed over the last decade to understand the essential genes in many bacterial and eukaryotic species^2,3^. TIS has helped the scientific community to understand genes involved in responses to a wide range of stress conditions and has enabled identification of many novel susceptibility and survival mechanisms^1^.

The first step in TIS methods is the generation of a transposon mutant library in which the resulting pool of mutants contains many random transposon insertions in all genes (except essential genes) of the target organism at multiple positions^4^. The location of transposon insertions is identified by sequencing from the transposon insertion site into adjacent regions of the target genome^5^. Saturated mutant libraries can provide very high resolution coverage of the genome allowing the importance of regions within genes to be identified as well as simply a role for the gene itself^6,7^.

To identify the repertoire of genes essential for responses to a particular stress condition, the transposon mutant library is grown under that stress condition (test) and in the absence of that stress (control). Differences in the prevalence of transposon mutants within the pools are compared between the stress condition of interest and the control^6^. Mutants with an advantage following exposure to the stress condition will proliferate while those that are disadvantaged will become depleted. Comparison of the total number of inserts at each site between control and condition experiments indicates loci of interest^8^. The DNA of mutant libraries is sequenced, using a customised protocol, [e.g. transposon-directed insertion site sequencing (TraDIS), transposon sequencing (Tn-seq), high-throughput insertion tracking by deep sequencing (HITS) and insertion sequencing (INSeq)], from the transposon reading into the adjacent genome to identify the location of transposon insertions^5,9–11^. These TIS methods use different transposon-genome junction enrichment techniques including restriction enzyme digestion, (e.g. MmeI), or the use of customised primer annealing followed by high-throughput sequencing of the resulting libraries^5,12^. These transposon based methods all use short-read sequencing, and the majority of them use either Illumina or Ion torrent sequencing platforms^13^.

**Figure 1:**
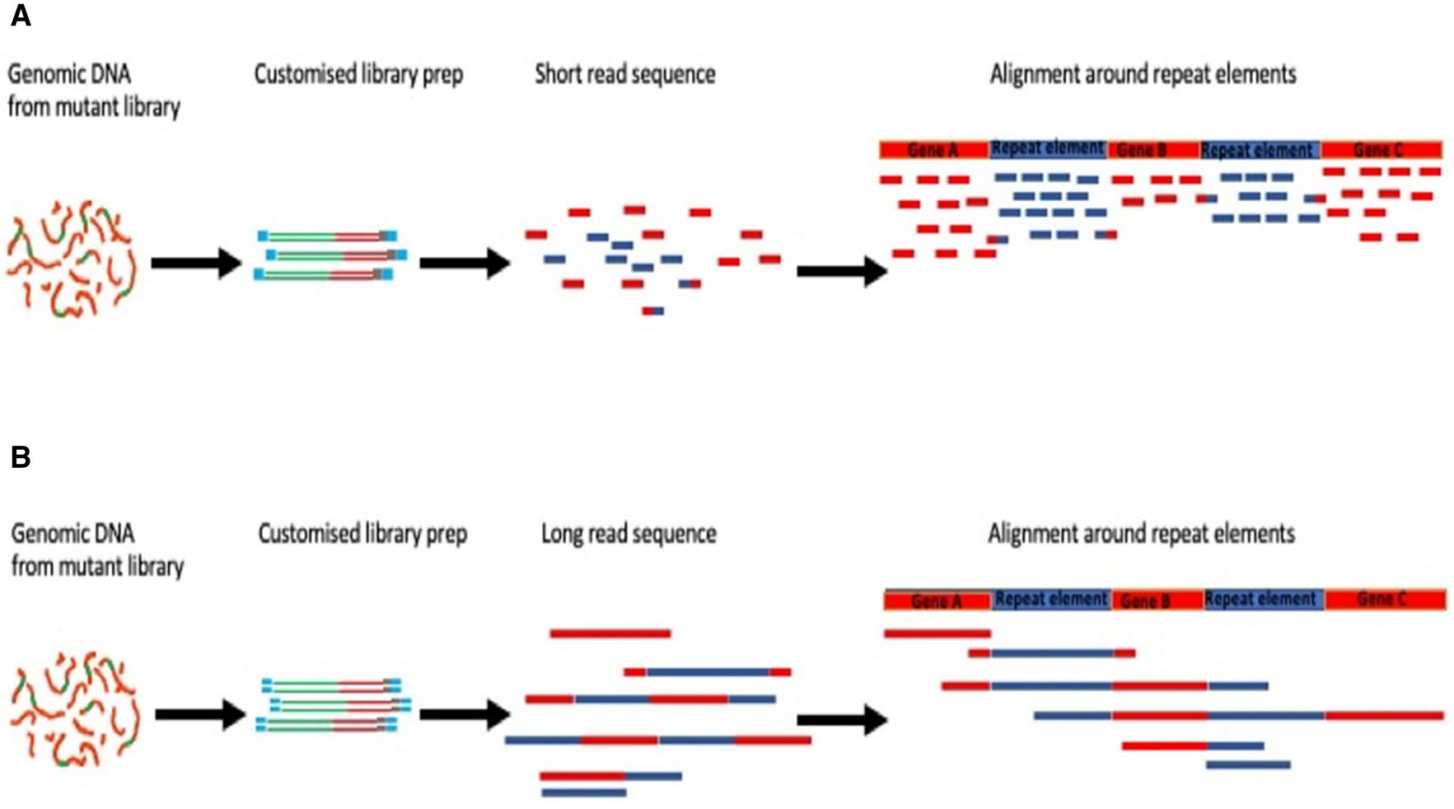
Mapping of transposon directed insertion site sequences to repeated nucleotide sequences in a reference genome. Panel (**A**) depicts the steps from genomic DNA to transposon directed insertion site sequencing and alignment of short-reads, indicating that short-reads cannot be mapped uniquely to repeated sequences. Panel (**B**) depicts the steps from genomic DNA to transposon directed insertion site sequencing and alignment of long-reads, indicating that long-reads resolve the mapping challenge across repeated sequences.

Sequence reads will not map uniquely to repeat sequences within a reference genome that are longer than the nucleotide sequence reads themselves (Fig. 1)^14^. The sequence read length generated by Illumina-based approaches is around 300 base pairs (for a single read), and so reads cannot map uniquely to repeat-sequence motifs that are longer than these reads^15^. Examples of long repeat regions in *E. coli* include transposase insertion sequences, which are usually over 600 bp, and the several copies of the ribosomal RNA operons, which are over 5 kb^16^. The repeat regions are even a greater challenge in eukaryotic genomes which are much larger and have numerous repeating genetic elements. Many repeating elements are of evolutionary importance, and/or have roles in replication, recombination, transcription and genome rearrangement^17^. Thus, transposon insertions into different copies of a repeating element may have different phenotypic effects. Therefore, determining the unique locations of transposon insertion sites in repeating elements is essential for formulating meaningful hypotheses from TIS data.

Here, we describe LoRTIS (Long-Read Transposon Insertion-site Sequencing), which overcomes the limitations resulting from using short-read nucleotide sequences. In LoRTIS, as with other TIS methods, we have used the known sequences of the transposon as an anchor point from which to generate DNA fragments by amplification of DNA into the unknown transposon insertion sites for sequencing. However, in LoRTIS, by modifying this amplification step, much longer DNA fragments are generated, from which long sequence reads are possible using a MinION nanopore sequencer (Oxford Nanopore)^18^. The resulting long-reads are then aligned with the nucleotide sequence of a reference genome, and the locations identifying the precise positions of the transposon insertions sites determined. The longer reads can span repeat sequences and read into adjacent unique sites. The long-reads generated by LoRTIS were able to locate transposon insertion sites uniquely within repeating sequence elements of the ribosomal RNA operons, which are approximately 5 kb. In addition, we incorporated different nucleotide sequence identifiers (indexes) into replicates to demonstrate that multiple samples can be sequenced on a single flow cell. Thus, LoRTIS can identify hundreds of thousands of transposon insertion sites uniquely and overcomes the limitation of short-reads generated by other TIS methods.

## Results

DNA was prepared for nucleotide sequencing using both the new LoRTIS method and the previously described TraDIS-*Xpress* protocols in duplicate, from DNA extraction to the generation of nucleotide sequence reads. This enables a comparison of the reproducibility within each method and also to compare the LoRTIS data against that generated using TraDIS-*Xpress.* For the first LoRTIS replicate, 8.7 million nucleotide sequence reads were generated, of which 4.2 million (48%) included transposon-specific sequences, whilst for the second replicate 7.6 million of 14.2 million reads (54%) had transposon-specific sequences. These data demonstrate that this novel method successfully enriched for transposon-genome junctions. The read lengths ranged from 300 bp to over 13,000 bp, averaging more than 1200 bp (Supplementary Table 1).

### Reproducibility of LoRTIS and comparison with TraDIS-*Xpress*

**Figure 2:**
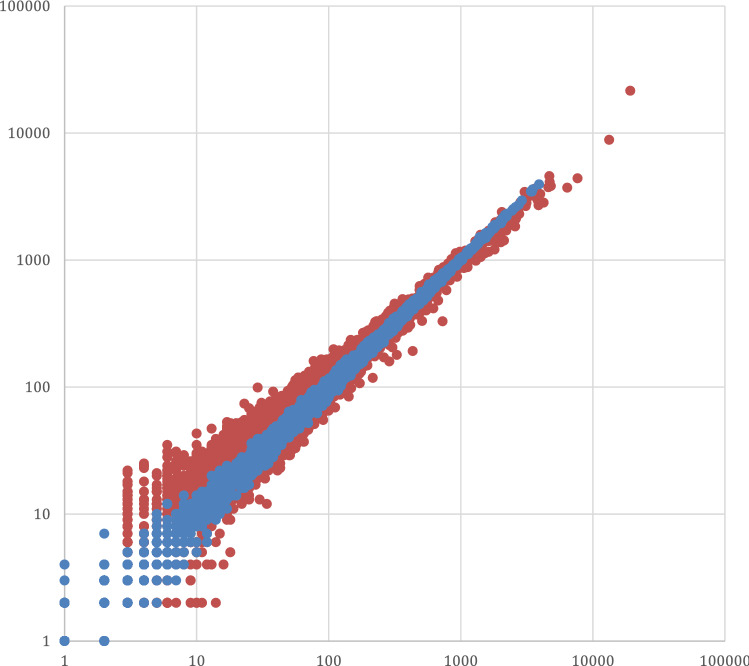
Scatter plot of the number of sequence-reads per gene using TraDIS-*Xpress* and LoRTIS. Each point on the chart represents a gene. For each gene, the number of sequence-reads mapped in replicate 1 is plotted against the number of reads mapped to the gene in replicate 2. Red points represent the scatter chart of LoRTIS results and blue points that of TraDIS-*Xpress* results. The strong correlation found indicates the reproducibility of each method.

**Figure 3:**
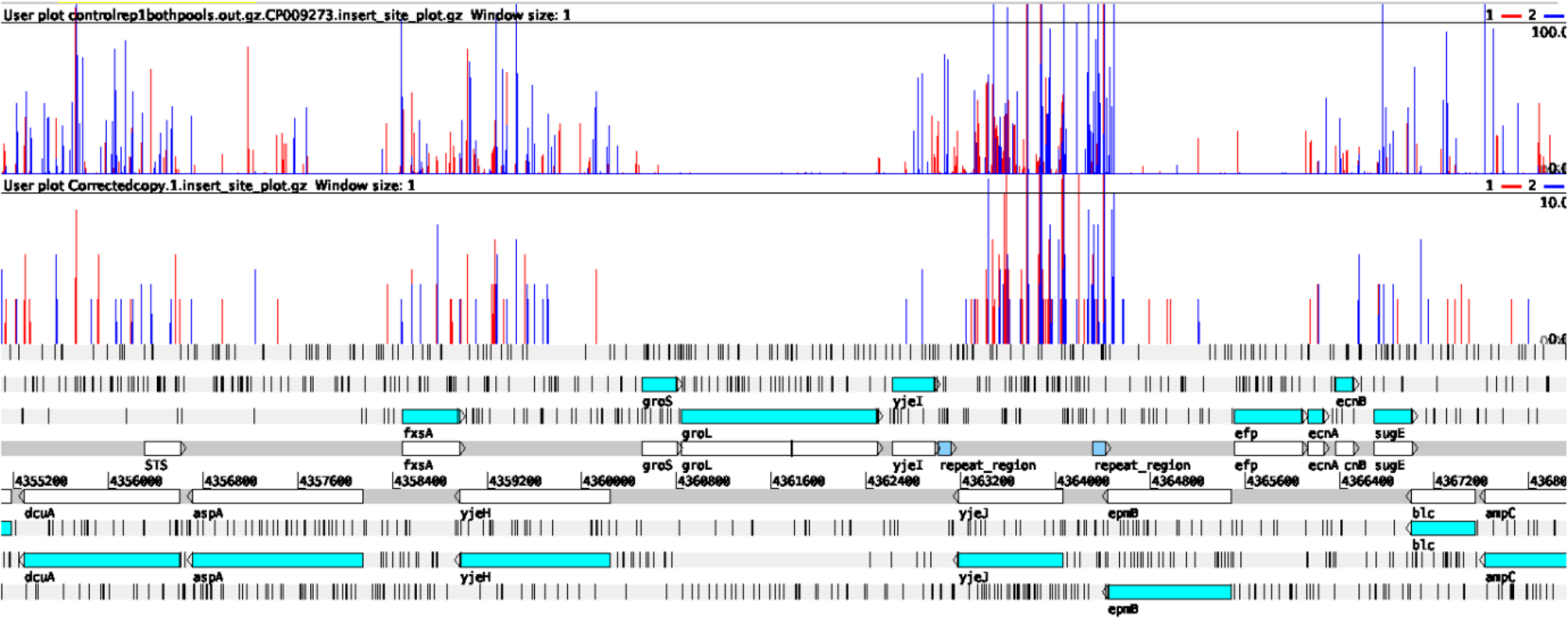
Comparison of transposon insertions sites identified using TraDIS-*Xpress* and LoRTIS. A genetic map of the relative gene positions is shown at the bottom of the panel. White arrowhead boxes represent the position of genes and blue arrowhead boxes represent encoded proteins from the genetic code. Above this, each row of vertical red or blue lines indicates the position of reads that mapped to the forward strand or reverse strand, respectively. The height of the bar represents the relative number of reads mapped to each position. The top row shows TraDIS-*Xpress* data, and the bottom row shows LoRTIS data, demonstrating the similarity between data generated by the two different methods.

The number of nucleotide sequence reads that mapped to each gene was determined, and comparison between these values for replicate 1 and replicate 2 in a scatter chart demonstrated the reproducibility of the LoRTIS method (Fig. 2). Comparing reads-per-gene generated from LoRTIS with data from TraDIS-*Xpress* highlights the similarity using the two different methods (Fig. 2). The Spearman correlation coefficient between the LoRTIS and TraDIS-*Xpress* data sets was 0.93. The distribution of mapped sequence reads also showed similarity in their positions and numbers between the two methods, indicating accurate calling of transposon insertion sites by LoRTIS (Fig. 3).

### Identification of candidate essential genes

During transposon mutagenesis for TIS experiments, mutants with transposon insertions in essential genes do not grow. Therefore, assuming sufficient transposon mutants and nucleotide sequence reads are generated to avoid stochastic areas of low coverage, TIS data should include relatively few sequence reads that match within essential genes. However, if the data includes insufficient sequence reads, leading to some genes being missed, then these will appear to be essential even when they are not, and so accurate calling of essential genes requires sufficient data to overcome this. Thus, an ideal control for the quality of TIS data is a clear demonstration that mapped reads are distributed across the entire genome, and enough data is generated to distinguish where very few or no reads map within known essential genes.

The LoRTIS data shown here not only resulted in sequence reads that were mapped across the entire genome but also demonstrated an absence of reads that mapped within many putative essential genes identified using TraDIS-*Xpress*. As an example, the similarity in distribution of mapped LoRTIS and TraDIS-*Xpress* generated reads across a short section of the genome is illustrated in Fig. 3. No sequence reads mapped to the candidate essential genes *groS* and *groL*, whereas there was an abundance of reads that mapped to the *dcuA*, *fxsA*, *yjeH* and *yjeJ* genes using both LoRTIS and TraDIS-*Xpress*, confirming that LoRTIS was at least equal to TraDIS-*Xpress* in this respect.

**Figure 4:**
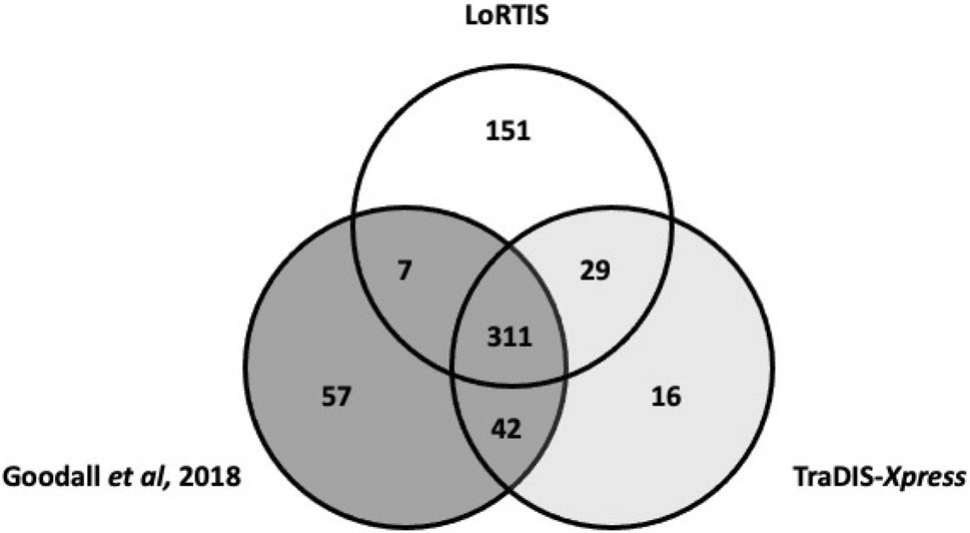
Putative essential genes identified using three different transposon insertion sequencing methods. Venn diagram showing the number of putative essential genes identified from three different transposon insertion sequencing sources: LoRTIS, TraDIS-*Xpress* and TraDIS^7^. Of these, 311 putative essential genes were common to all three data sets.

A list of putative essential genes generated from our LoRTIS data was also compared with lists derived from TraDIS-*Xpress* data and conventional TraDIS data from another group^6,7^. These reference data sets were selected for comparison because they were generated from the same strain of *E. coli* (BW25113). TraDIS-*Xpress* and TraDIS data were produced using the Illumina platform for sequence generation, while LoRTIS used nanopore sequencing. Comparisons of putative essential genes showed that 311 essential genes identified were common to all three methods (Fig. 4; Supplementary Table 2). Figure 4 depicts the putative essential genes identified using each method and their relative distribution. Of 398 putative essential genes that were identified by our TraDIS-*Xpress* data, 340 (85%) were also identified by LoRTIS.

### Advantages of long sequence reads in mapping transposon insertion sites within regions of repeating nucleotide sequence

**Figure 5:**
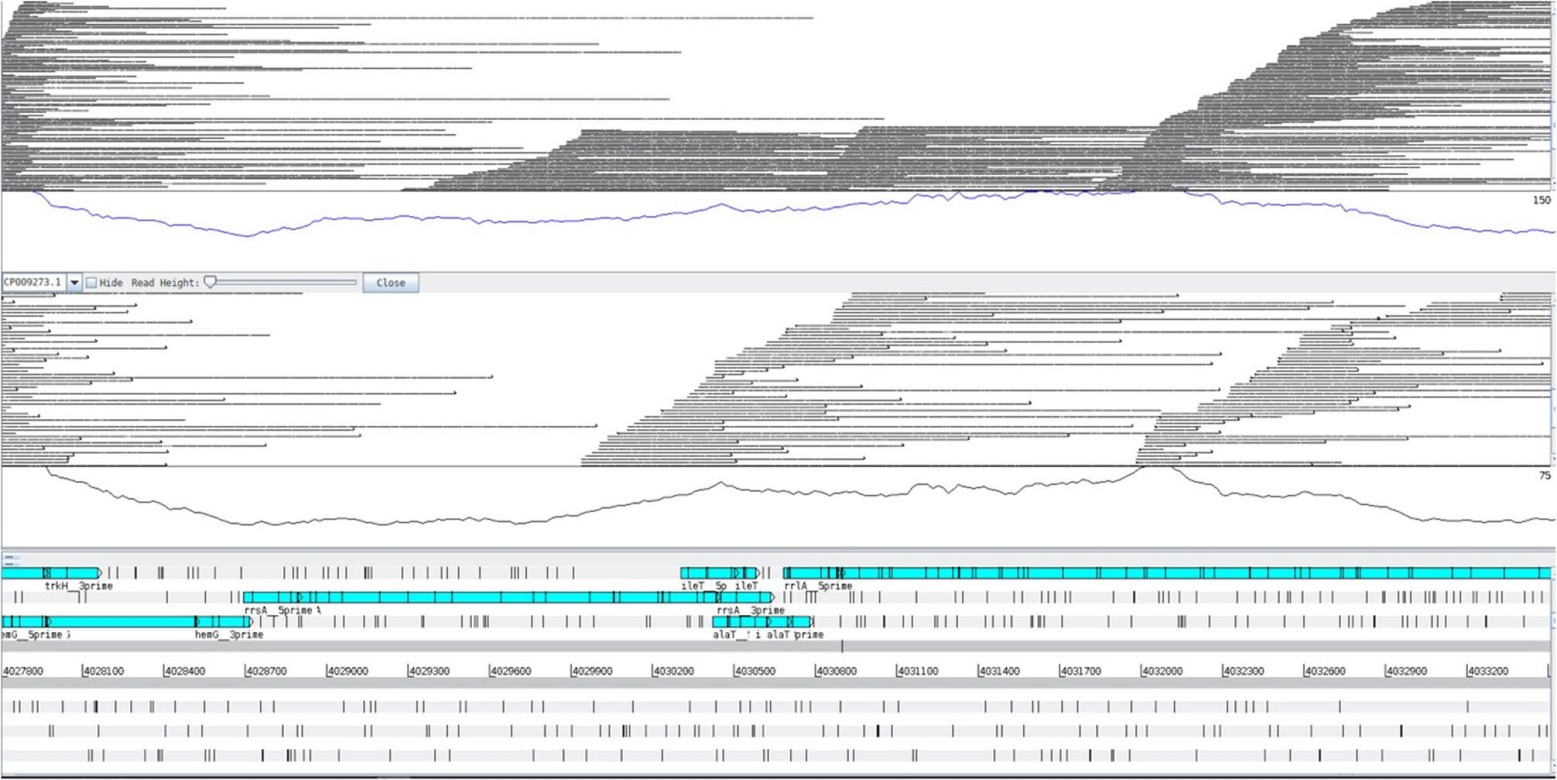
Long-reads mapped uniquely to a single copy of a repeating ribosomal RNA gene cluster of over 5 kb. In the *E. coli* genome, the longest repeating nucleotide sequences are those of the ribosomal RNA operons, of which there are seven copies, each of over 5 kb in length. These are, therefore, a good test of the ability of LoRTIS to identify insertion sites uniquely within long repeating sequences. A genetic map of the relative rRNA gene positions is shown at the bottom of the panel as blue arrowhead boxes. Above this, each panel depicts the long-reads, each shown as a fine horizontal line, that map uniquely within this repeat element.

Long-reads are particularly helpful in mapping to unique sites in the genome when the genome size of the organism is large or there are repeat elements. LoRTIS can produce long-reads that map across repeat elements and into unique regions of the genome enabling us to identify transposon insertions. In *E. coli* BW25113, there are seven ribosomal RNA operons; each is over 5 kb in length and contains two ribosomal RNA genes that are highly conserved. Reads generated by TraDIS-*Xpress* could not be mapped to these operons uniquely while reads generated by LoRTIS could. Although most of the reads generated in this study were between 0.3 and 2 kb in length, they mapped uniquely. This was because either the reads spanned regions of polymorphisms within the repeated elements, or the reads extended into unique flanking nucleotide sequences (Fig. 5).

Another set of repeat elements in *E. coli* are the *ins* loci (*insA, insB, insH*) that have more than one copy of genes distributed across the genome^16^. Transposon insertions have been reported in these *ins* loci but again could not be mapped to a given copy with certainty using short-reads. In our LoRTIS data, there were more than 47,000 sequence reads that mapped to *ins* loci, of which ~ 22,000 mapped uniquely (47%) while in the TraDIS-*Xpress* data generated by short-read platform Illumina, out of 28,000 reads mapping to *ins* loci, only ~ 6500 mapped uniquely (17%) (Supplementary Table 3). These data demonstrate that LoRTIS long-reads can uniquely map reads across repeat elements more effieciently than TraDIS-*Xpress*.

### Multiplexing of LoRTIS experiments

**Figure 6:**
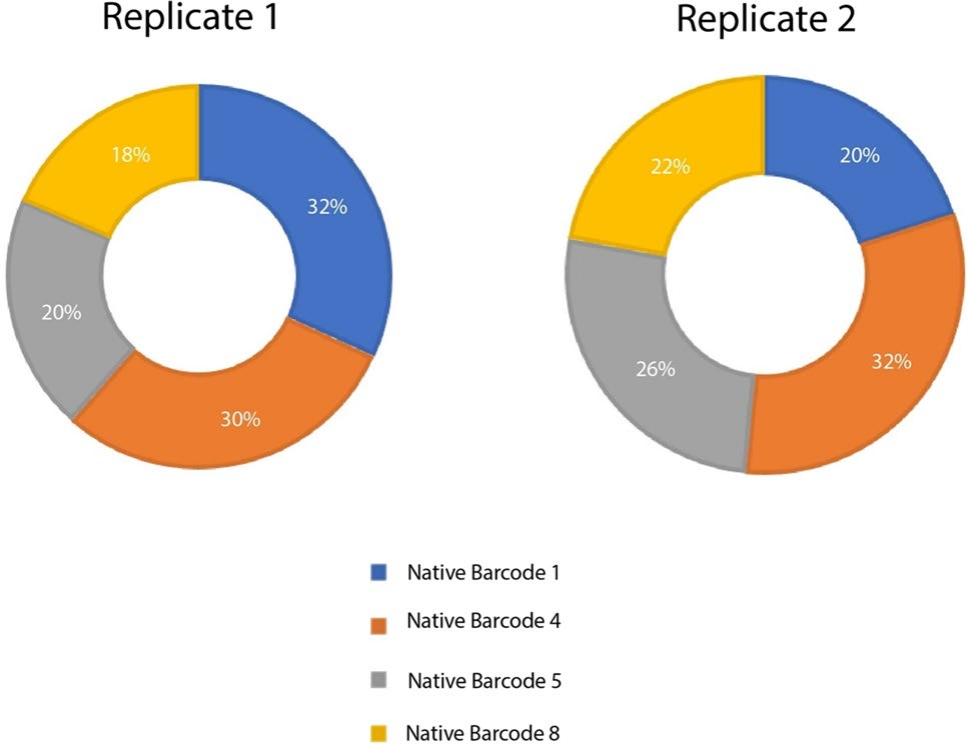
Native Barcode reads demultiplexed from LoRTIS data. The circular plots represent the total number of transposon specific sequences containing reads that were demultiplexed from LoRTIS data. Replicate 1 had reads from all four Native Barcodes (NBDs): 32% of reads were from NBD1 (blue), 30% from NBD4 (brown), 20% from NBD5 (grey) and 18% from NBD8 (yellow). Replicate 2 had reads from all four NBDs: 20% from NBD1 (blue), 32% from NBD4 (brown), 26% from NBD5 (grey) and 22% from NBD8 (yellow).

A unique sequence identifier (barcode) can be added to DNA fragments of a sample during the sequencing library preparation step that allows different samples to be combined and sequenced on a single flow cell (multiplexed) and after sequencing, reads from each sample can be separated from the pool based on the barcode (demultiplexed). Oxford Nanopore uses 24 bp sequences to assign a unique identifier to each sample; these are called Native Barcodes (NBD), and 96 NBD are available. We used four of these NBD to multiplex our LoRTIS DNA fragment preparations. Of the sequence reads from our LoRTIS experiment, 94% and 84% were demultiplexed into these unique NBD in replicate 1 and replicate 2, respectively. Although each NBD produced different numbers of reads, there was no bias observed in using any particular NBD (Fig. 6). This confirms that LoRTIS can successfully incorporate multiplexing of different experimental samples on a single MinION flow cell.

## Discussion

TIS methods offer a robust approach for comparing the fitness of libraries of mutants in parallel, and to probe genotype–phenotype associations^19^. They have been used to understand the mechanisms of antibiotic susceptibility, to evaluate fitness mechanisms in a range of condition-specific environments^2^ and to determine the genetic basis of mutants that have been physically sorted based on an unusual phenotype^20^. Most reported TIS methods employ Illumina nucleotide sequencing technology to generate sequence reads from transposons and find their insertion sites. Whilst this technology is suited to TIS as it generates millions of nucleotide sequence reads, contributing to experimental accuracy and enabling the sequencing of several experiments to be conducted in parallel^21^, it comes with a number of technical challenges. One of these is that the Illumina platform requires nucleotide sequence diversity to successfully register the different clusters of DNA molecules from which the nucleotide sequences are read^5^. Using TIS all the sequence reads start with the same nucleotide sequence of the transposon, leading to failure in cluster registration and therefore the whole sequencing run. This has been overcome in the past either by using ‘dark cycles’ or by addition of other DNA to a sequencing run to create sequence diversity at each sequencing cycle^6,7^. This, however, results in a significant proportion (up to 50%) of sequencing capacity being used to enable cluster registration rather than in the generation of useful TIS sequence data^6^.

Another drawback of using standard short-read TIS methods is the inability to map reads uniquely to repeated nucleotide sequences whose repeat units are longer than the sequence reads^14^. In bacteria repeat regions are common and more than 10% of the genome is comprised of repeated elements; in eukaryotes it is often more than 50%^22^. Therefore, conventional TIS methods using short-read sequencing will be of limited value in eukaryotes. With LoRTIS these drawbacks do not exist, as nanopore MinION flow cells do not perform any cluster registration and all reads can start with the same nucleotide sequence. In addition, LoRTIS generates much longer sequence reads so that transposon insertion sites can be mapped unambiguously and uniquely, even within repeating nucleotide sequences of the target genome^23^.

In the results section, we described how long sequence reads generated by LoRTIS were able to uniquely map to rRNA operons (Fig. 5). There are seven rRNA operons in *E. coli* BW25113, each more than 5 kb in length and highly conserved. As TraDIS-*Xpress* uses short-reads it cannot map uniquely to these regions and so insert sites cannot be indentified uniquely. In contrast, LoRTIS has a range of read lengths from 300 bp to 13 kb and was able to identify transposon insertions uniquely within the repeated nucleotide sequences of the rRNA operons. This unique mapping was achieved because the long-reads either extended into unique flanking sequences, or spanned sufficient polymorphism within the repeated sequences to allow a specific match. Our current method may also be applied to large eukaryotic genomes where short sequence reads are insufficient to map transposon-genome junctions uniquely to the cognate genome, which can include numerous repeating elements, such as Long Interspersed Nuclear Elements (LINEs) and Short Interspersed Nuclear Elements (SINEs)^24^.

In the past, long-read nucleotide sequencing techniques have been used to study transposon insertion sites in prokaryotes, but only when coupled with whole genome sequencing (WGS)^25,26^. However, identifying the location of tens of thousands of mutants using this approach may not be practical. CRISPR/cas has also been used to capture transposon-genome junctions followed by long-read sequencing but this method also lacks efficiency^26^. LoRTIS applies enrichment steps to capture the transposon-genome junctions and allows parallel sequencing of millions of captured transposon insertion sites which gives efficiency comparable to current TIS approaches using Illumina sequencing.

In LoRTIS, we used streptavidin Dynabeads to enrich and select for transposon-genome junctions and longamp Taq polymerase PCR to produce long DNA fragments. Dynabead capture of transposon gene junctions is an essential part of the protocol as PCR alone failed to capture these junctions efficiently (data not shown). Apart from the advantage of enabling the mapping of insertion sites uniquely within repeating nucleotide sequences, LoRTIS may also be relatively economical, although comparisons with existing short-read technologies are not straightforward (Supplementary Table 4).

In conclusion, LoRTIS works like other TIS methods e.g. TraDIS-*Xpress*, but provides an effective and flexible approach to use nanopore sequencing for TIS experiments which can be run at various scales, without expensive sequencing machines and generates data that offers the significant advantage of being able to unambiguously map a greater range of insertion sites.

## Methods

### DNA extraction and experimental setup

The *Escherichia coli* strain BW25113 large transposon mutant library described by Yasir et al. was used in this study^6^. Genomic DNA was extracted using the Quick-DNA Fungal/Bacterial 96 Kit (Zymo Research).

LoRTIS and TraDIS-*Xpress* samples were prepared in duplicate, from DNA extraction to sequencing library preparation. For LoRTIS each replicate was sequenced on a separate flow cell, so Native Barcodes (NBD) were added to each replicate to test the multiplexing capability of the LoRTIS method. NBD details are shown in Supplementary Table 5.

### Preparation of DNA fragments for LoRTIS and TraDIS-*Xpress*

For LoRTIS sequencing library preparation,1 μg of genomic DNA was tagmented using an Illumina compatible MuSeek library preparation kit (Thermo Fisher Scientific) and the tagmented DNA was cleaned using 0.5× volume of AMPure XP magnetic beads (Beckman-Coulter). LongAmp *Taq* DNA polymerase (New England Biolabs, UK) was used with biotinylated oligonucleotide primers (Supplementary Table 5) that hybridise specifically within the transposon to amplify DNA fragments containing transposon insertion junctions (Supplementary Table 6 PCR1a). A second primer that anneals to MuSeek adapters (‘IonTMu-02’) was used to generate biotinylated double-stranded PCR products containing transposon-genome junctions (Supplementary Table 6 PCR1b). These PCR products were enriched using a Dynabeads™ kilobaseBINDER™ kit (Thermo Fisher Scientific). A nested PCR reaction was carried out using NEB LongAmp *Taq* polymerase to generate DNA fragments with Oxford Nanopore multiplexing adapters that lacked biotin (Supplementary Table 6 PCR2). The PCR products from this reaction were purified using 0.5× volume of AMPure XP magnetic beads (Beckman-Coulter) and were end-repaired, then nanopore sequencing adapters were ligated according to the Oxford Nanopore protocol.

**Figure 7:**
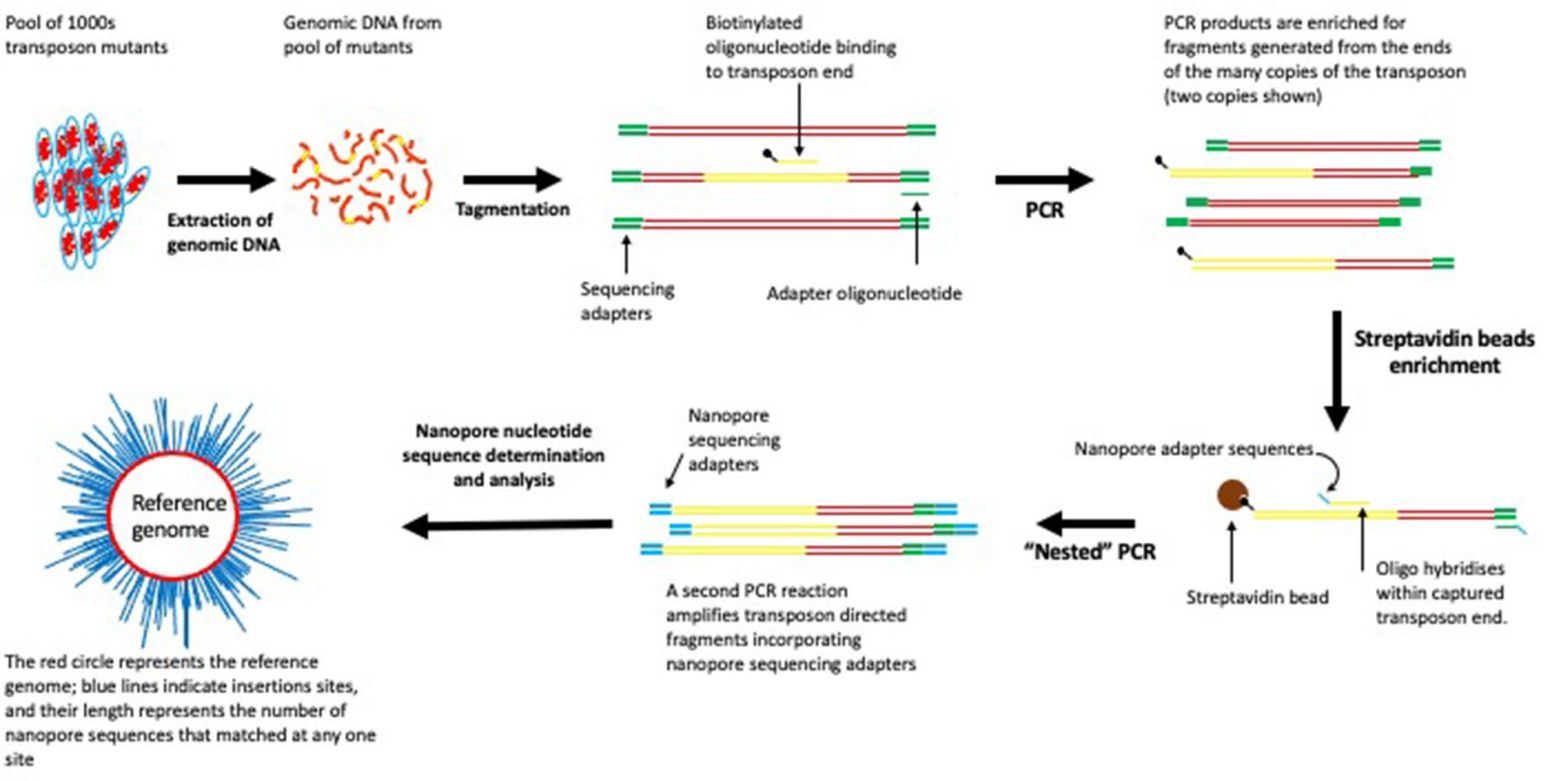
An overview of LoRTIS sequencing library preparation and alignment. The gDNA was extracted from a high-density transposon insertion library containing multiple insertions in every non-essential genomic locus. The gDNA was tagmented and transposon junctions were amplified using one biotinylated primer followed by another step using two primers for amplification. Biotinylated DNA fragments were selected using streptavidin beads and nested PCR was done to add sequencing primers. The library was sequenced using nanopore and the reads mapped to the reference genome using Bio-LoRTIS software (see “Materials and methods”).

For nucleotide sequencing by LoRTIS, the resulting DNA fragments were loaded onto an Oxford Nanopore MinION flowcell and sequenced for up to 48 h (Fig. 7). Using the LoRTIS protocol, nanopore long-read nucleotide sequence data were generated from two separate runs on an Oxford Nanopore flow cell to generate two replicate data sets that could be compared for reproducibility.

For comparison, DNA fragments were also prepared for generation of nucleotide sequence reads using TraDIS-*Xpress* methods as described previously by Yasir et al. and sequenced using the Illumina platform^6^.

### Bioinformatics

FAST5 format data from MinION was processed using Guppy Basecalling software (version 3.6.0) running in High Accuracy Calling (HAC) mode and using GPUs on the Quadram Institute cloud infrastructure. The resulting sequence data in FASTQ format were demultiplexed using QCat (version 1.1.0) and those reads containing transposon nucleotide sequences were identified, trimmed and retained, again using QCat. The nucleotide sequence reads were then located to the BW25113 reference genome (CP009273) using Bio-TraDIS (version 1.4.1)^27^ and Bio-LoRTIS software (version 0.0.5)^28^ in a similar way as for short-read data, except that steps to remove the transposon sequences were skipped and Minimap2 (version 2.17-r941)^29^ was substituted in the place of SMALT. Results from BioTradis, including plot files, were outputted in the same format as for short-read data.

The insertion patterns at candidate loci were inspected visually using Artemis (version 18.1.0) which was also used to capture images for figures^30^. Gene essentiality was determined using tradis_essentiality.R from BioTradis, and scatter charts were plotted to determine reproducibility using Microsoft Excel after normalising for numbers of reads in each replicate. The correlation between TraDIS-*Xpress* and LoRTIS data was calculated using Spearman's rank correlation coefficient.

## Supplementary Information

Supplementary Tables.

## Data Availability

All sequence data have been deposited with the European Bioinformatics Institute (EBI). The overall project accession number is E-MTAB-11351.
