# Supplementary material for: Long-read sequencing for identification of insertion sites in large transposon mutant libraries
Source: Sci Rep. 2022 Mar 3;12:3546. doi: 10.1038/s41598-022-07557-x (PMC8894413; doi:10.1038/s41598-022-07557-x)
Supplement: Supplementary file 1 — Supplementary Tables. [file 41598_2022_7557_MOESM1_ESM.docx]

**Supplementary Material**

**Long read sequencing for identification of insertion sites in large transposon mutant libraries**

**Muhammad Yasir^1^, A. Keith Turner^1^, Martin Lott^1^, Steven Rudder^1^, David Baker^1^, Sarah Bastkowski^1^, Andrew J. Page^1^, Mark A. Webber^1,2^, Ian G. Charles^1,2^**

^1^Quadram Institute Bioscience, Rosalind Franklin Road, Norwich, NR4 7UQ.

^2^Norwich Medical School, Norwich Research Park, Colney Lane, Norwich, NR4 7TJ.

^2^University of East Anglia, Norwich Research Park, Norwich, NR4 7TJ.

**Supplementary Table 1. Details of LoRTIS (Long Read Transposon Insertion site Sequencing) reads**

| **Parameter** | **Replicate 1** | **Replicate 2** |
| --- | --- | --- |
| Total number of reads | 8723591 | 14206871 |
| Number of reads with transposon-specific sequences | 4206713 | 7629034 |
| Percentage of reads with transposon-specific sequences | 48.2% | 53.7% |
| Number of reads mapped | 1822805 | 3064254 |
| Average read length before trimming | 1325.33 bp | 1147.88 bp |
| Average read length after trimming | 562.12 bp | 490.22 bp |
| Longest Nanopore read | 20570 bp | 25053 bp |
| Longest read mapped | 13954 bp | 12465 bp |

**Supplementary Table 2**

**Putative essential genes of *E. coli* BW25113 using different TIS methods**

| All three | Nanotradis | Yasir  et al.,  2020 | Goodall  et al., 2018 | NanoTraDIS and  Yasir et al., 2020 | NanoTraDIS and  Goodall et al., 2018 | Yasir et al.,  2020 and  Goodall  et al., 2018 |
| --- | --- | --- | --- | --- | --- | --- |
| rpsT | ileV | argW | cydB | mtn | argU | thyA |
| ribF | rrfH | yciM | *adapF* | tff | cydX | dnaC |
| ileS | aspU | rpmG | *fdx* | ppiB | tonB | hemH |
| lspA | gmhA | ratA | glyA | ybeD | yefM | priB |
| ispH | thrW | nuoI | iraM | glnW | hda | lpxB |
| dapB | ykfN | dnaK | rpe | glnU | rnc | plsB |
| folA | ykgS | rlmE | safA | cmk | rbfA | ribC |
| ftsL | insB1 | purA | tktA | rdlA | 7 | lepB |
| ftsI | ptwF | rimP | ycaR | rdlC |  | def |
| murE | insE1 | ptsH | ydaE | tyrV |  | ftsW |
| murF | yaiY | nuoM | ydaS | tyrT |  | hemB |
| mraY | xseB | rpiA | ydcD | cysB |  | wzyE |
| murD | glnV | icd | yddL | sokB |  | ispD |
| murC | metU | pgpB | ydfO | ydfA |  | ftsQ |
| ftsA | metT | nuoN | ydhL | valV |  | eno |
| lpxC | kdpF | dnaQ | yedN | rydB |  | coaA |
| aceF | sdhC | 16 | ygeF | ydiH |  | degS |
| lpd | lysT |  | ygeG | azuC |  | ubiX |
| can | lysW |  | ygeN | crr |  | folD |
| map | valZ |  | yjbS | hscB |  | folP |
| rpsB | lysY |  | ykfM | micA |  | sucB |
| pyrH | lysZ |  | ymfE | rnpB |  | tsaC |
| frr | lysQ |  | yncH | rpmJ |  | yffS |
| cdsA | mntS |  | yqeL | rpmE |  | hipB |
| rseP | rybB |  | *alsK* | holC |  | ftsH |
| fabZ | croE |  | *bcsB* | ubiF |  | secF |
| dnaE | ldrA |  | *mazE* | nuoH |  | birA |
| accA | rdlB |  | *chpS* | crp |  | rnpA |
| proS | ldrC |  | *entD* | ssrA |  | tusE |
| ribE | sapC |  | *minD* | 29 |  | lptA |
| ispA | pspD |  | *waaU* |  |  | yobI |
| ffs | ogt |  | *tnaB* |  |  | rpsR |
| dnaX | fnrS |  | *tdcF* |  |  | priA |
| adk | rcbA |  | *yabQ* |  |  | psd |
| lpxH | kilR |  | yafF |  |  | yqcG |
| cysS | ydaF |  | *yagG* |  |  | nusB |
| lipA | ydcA |  | *ydiL* |  |  | dcd |
| mrdB | ydcX |  | *mqsA* |  |  | yejM |
| mrdA | yddM |  | yhbV |  |  | minE |
| nadD | marB |  | *yhhQ* |  |  | ihfA |
| holA | relE |  | *yibJ* |  |  | sucA |
| lptE | valW |  | ubiJ |  |  | polA |
| ybeY | ydhX |  | *yqgD* |  |  | 42 |
| leuW | sufA |  | *rsmI* |  |  |  |
| fldA | menI |  | mlaB |  |  |  |
| infA | ydiE |  | *kdsC* |  |  |  |
| lolA | ydiZ |  | *folK* |  |  |  |
| serS | zwf |  | ftsE |  |  |  |
| msbA | yecJ |  | *ftsK* |  |  |  |
| kdsB | glyW |  | *ftsN* |  |  |  |
| mukF | fliJ |  | *ftsX* |  |  |  |
| mukE | dsrA |  | *ribB* |  |  |  |
| mukB | rseX |  | *rne* |  |  |  |
| asnS | asnU |  | secM |  |  |  |
| fabA | asnV |  | *spoT* |  |  |  |
| serT | yoeF |  | yceQ |  |  |  |
| lpxL | hisL |  | *lptC* |  |  |  |
| rpmF | ibsA |  | 57 |  |  |  |
| fabH | sibB |  |  |  |  |  |
| fabG | ibsB |  |  |  |  |  |
| acpP | cyaR |  |  |  |  |  |
| tmk | micF |  |  |  |  |  |
| holB | ypaA |  |  |  |  |  |
| lolC | nuoA |  |  |  |  |  |
| lolE | yfcZ |  |  |  |  |  |
| mnmA | pawZ |  |  |  |  |  |
| cohE | insL1 |  |  |  |  |  |
| pth | alaW |  |  |  |  |  |
| ispE | valU |  |  |  |  |  |
| hemA | valY |  |  |  |  |  |
| prfA | lysV |  |  |  |  |  |
| prmC | eutN |  |  |  |  |  |
| trpL | ypfM |  |  |  |  |  |
| ribA | iroK |  |  |  |  |  |
| yciS | glmY |  |  |  |  |  |
| ymiB | rrfG |  |  |  |  |  |
| fabI | rrlG |  |  |  |  |  |
| ttcC | gltW |  |  |  |  |  |
| relB | psaA |  |  |  |  |  |
| dicA | argZ |  |  |  |  |  |
| pdxH | argY |  |  |  |  |  |
| rnt | argV |  |  |  |  |  |
| pheT | ygdG |  |  |  |  |  |
| pheS | metZ |  |  |  |  |  |
| pheM | ssrS |  |  |  |  |  |
| rplT | ibsC |  |  |  |  |  |
| rpmI | ibsE |  |  |  |  |  |
| infC | ileX |  |  |  |  |  |
| thrS | psrO |  |  |  |  |  |
| nadE | yhcO |  |  |  |  |  |
| gapA | rrfF |  |  |  |  |  |
| tsaB | thrV |  |  |  |  |  |
| aspS | rrfD |  |  |  |  |  |
| leuZ | rrlD |  |  |  |  |  |
| cysT | alaU |  |  |  |  |  |
| pgsA | ileU |  |  |  |  |  |
| rplY | rrsD |  |  |  |  |  |
| gyrA | tusB |  |  |  |  |  |
| ubiG | ryhB |  |  |  |  |  |
| nrdA | dinQ |  |  |  |  |  |
| nrdB | agrB |  |  |  |  |  |
| accD | gltU |  |  |  |  |  |
| fabB | rrfC |  |  |  |  |  |
| ligA | aspT |  |  |  |  |  |
| zipA | leuT |  |  |  |  |  |
| ptsI | ileT |  |  |  |  |  |
| dapE | alaT |  |  |  |  |  |
| dapA | spf |  |  |  |  |  |
| der | rrlB |  |  |  |  |  |
| hisS | tyrU |  |  |  |  |  |
| ispG | thrT |  |  |  |  |  |
| hscA | rrsE |  |  |  |  |  |
| iscU | gltV |  |  |  |  |  |
| iscS | rrlE |  |  |  |  |  |
| tadA | rrfE |  |  |  |  |  |
| acpS | yjaA |  |  |  |  |  |
| era | pmrR |  |  |  |  |  |
| rpoE | pheU |  |  |  |  |  |
| pssA | glyV |  |  |  |  |  |
| rluD | ryjB |  |  |  |  |  |
| bamD | rrlA |  |  |  |  |  |
| rplS | rrlH |  |  |  |  |  |
| trmD | rrlC |  |  |  |  |  |
| rimM | nuoG |  |  |  |  |  |
| rpsP | nuoC |  |  |  |  |  |
| grpE | rrsC |  |  |  |  |  |
| nadK | rrsA |  |  |  |  |  |
| serV | glnA |  |  |  |  |  |
| csrA | nuoF |  |  |  |  |  |
| alaS | wcaI |  |  |  |  |  |
| ispF | ackA |  |  |  |  |  |
| ftsB | purR |  |  |  |  |  |
| pyrG | valT |  |  |  |  |  |
| lgt | gnsA |  |  |  |  |  |
| prfB | ldrB |  |  |  |  |  |
| fbaA | ydfZ |  |  |  |  |  |
| pgk | serU |  |  |  |  |  |
| metK | yohO |  |  |  |  |  |
| yqgF | rrsG |  |  |  |  |  |
| plsC | metY |  |  |  |  |  |
| parE | istR |  |  |  |  |  |
| folB | ilvX |  |  |  |  |  |
| rpsU | rrsB |  |  |  |  |  |
| dnaG | gltT |  |  |  |  |  |
| rpoD | fepG |  |  |  |  |  |
| rpsO | btuE |  |  |  |  |  |
| leuU | nuoL |  |  |  |  |  |
| glmM | sapF |  |  |  |  |  |
| obgE | yciA |  |  |  |  |  |
| rpmA | rrsH |  |  |  |  |  |
| rplU | yffQ |  |  |  |  |  |
| murA | 151 |  |  |  |  |  |
| rpsI |  |  |  |  |  |  |
| rplM |  |  |  |  |  |  |
| mreB |  |  |  |  |  |  |
| accB |  |  |  |  |  |  |
| accC |  |  |  |  |  |  |
| fmt |  |  |  |  |  |  |
| rplQ |  |  |  |  |  |  |
| rpsD |  |  |  |  |  |  |
| rpsK |  |  |  |  |  |  |
| rpsM |  |  |  |  |  |  |
| secY |  |  |  |  |  |  |
| rplO |  |  |  |  |  |  |
| rpmD |  |  |  |  |  |  |
| rpsE |  |  |  |  |  |  |
| rplR |  |  |  |  |  |  |
| rplF |  |  |  |  |  |  |
| rpsH |  |  |  |  |  |  |
| rpsN |  |  |  |  |  |  |
| rplE |  |  |  |  |  |  |
| rplX |  |  |  |  |  |  |
| rplN |  |  |  |  |  |  |
| rpsQ |  |  |  |  |  |  |
| rpmC |  |  |  |  |  |  |
| rplP |  |  |  |  |  |  |
| rpsC |  |  |  |  |  |  |
| rplV |  |  |  |  |  |  |
| rpsS |  |  |  |  |  |  |
| rplB |  |  |  |  |  |  |
| rplW |  |  |  |  |  |  |
| rplD |  |  |  |  |  |  |
| rplC |  |  |  |  |  |  |
| rpsJ |  |  |  |  |  |  |
| rpsG |  |  |  |  |  |  |
| rpsL |  |  |  |  |  |  |
| asd |  |  |  |  |  |  |
| waaA |  |  |  |  |  |  |
| coaD |  |  |  |  |  |  |
| rpmB |  |  |  |  |  |  |
| dut |  |  |  |  |  |  |
| gmk |  |  |  |  |  |  |
| dnaA |  |  |  |  |  |  |
| rpmH |  |  |  |  |  |  |
| glmS |  |  |  |  |  |  |
| trpT |  |  |  |  |  |  |
| argX |  |  |  |  |  |  |
| hisR |  |  |  |  |  |  |
| proM |  |  |  |  |  |  |
| ubiE |  |  |  |  |  |  |
| ubiB |  |  |  |  |  |  |
| hemG |  |  |  |  |  |  |
| murI |  |  |  |  |  |  |
| thrU |  |  |  |  |  |  |
| glyT |  |  |  |  |  |  |
| secE |  |  |  |  |  |  |
| nusG |  |  |  |  |  |  |
| rplK |  |  |  |  |  |  |
| rplA |  |  |  |  |  |  |
| rplJ |  |  |  |  |  |  |
| rplL |  |  |  |  |  |  |
| rpoB |  |  |  |  |  |  |
| hemE |  |  |  |  |  |  |
| ubiA |  |  |  |  |  |  |
| lexA |  |  |  |  |  |  |
| ssb |  |  |  |  |  |  |
| groS |  |  |  |  |  |  |
| groL |  |  |  |  |  |  |
| efp |  |  |  |  |  |  |
| rsgA |  |  |  |  |  |  |
| orn |  |  |  |  |  |  |
| tsaE |  |  |  |  |  |  |
| rpsF |  |  |  |  |  |  |
| valS |  |  |  |  |  |  |
| lptF |  |  |  |  |  |  |
| lptG |  |  |  |  |  |  |
| dnaT |  |  |  |  |  |  |
| holD |  |  |  |  |  |  |
| topA |  |  |  |  |  |  |
| leuS |  |  |  |  |  |  |
| bamA |  |  |  |  |  |  |
| gyrB |  |  |  |  |  |  |
| parC |  |  |  |  |  |  |
| yrfF |  |  |  |  |  |  |
| fusA |  |  |  |  |  |  |
| dxs |  |  |  |  |  |  |
| glnS |  |  |  |  |  |  |
| yidC |  |  |  |  |  |  |
| cydA |  |  |  |  |  |  |
| murJ |  |  |  |  |  |  |
| lysS |  |  |  |  |  |  |
| gltX |  |  |  |  |  |  |
| dnaB |  |  |  |  |  |  |
| purB |  |  |  |  |  |  |
| glmU |  |  |  |  |  |  |
| ffh |  |  |  |  |  |  |
| secA |  |  |  |  |  |  |
| infB |  |  |  |  |  |  |
| tilS |  |  |  |  |  |  |
| hemL |  |  |  |  |  |  |
| tyrS |  |  |  |  |  |  |
| folC |  |  |  |  |  |  |
| rho |  |  |  |  |  |  |
| cca |  |  |  |  |  |  |
| dfp |  |  |  |  |  |  |
| dxr |  |  |  |  |  |  |
| ubiH |  |  |  |  |  |  |
| lptD |  |  |  |  |  |  |
| ftsZ |  |  |  |  |  |  |
| ribD |  |  |  |  |  |  |
| mreC |  |  |  |  |  |  |
| murG |  |  |  |  |  |  |
| glyS |  |  |  |  |  |  |
| murB |  |  |  |  |  |  |
| lpxD |  |  |  |  |  |  |
| gpsA |  |  |  |  |  |  |
| metG |  |  |  |  |  |  |
| tsaD |  |  |  |  |  |  |
| trpS |  |  |  |  |  |  |
| dapD |  |  |  |  |  |  |
| lpxK |  |  |  |  |  |  |
| fabD |  |  |  |  |  |  |
| lolB |  |  |  |  |  |  |
| kdsA |  |  |  |  |  |  |
| ynbG |  |  |  |  |  |  |
| ydfB |  |  |  |  |  |  |
| lptB |  |  |  |  |  |  |
| rpoA |  |  |  |  |  |  |
| argS |  |  |  |  |  |  |
| rpoC |  |  |  |  |  |  |
| ygfZ |  |  |  |  |  |  |
| thiL |  |  |  |  |  |  |
| prs |  |  |  |  |  |  |
| hemC |  |  |  |  |  |  |
| rpoH |  |  |  |  |  |  |
| tsf |  |  |  |  |  |  |
| lpxA |  |  |  |  |  |  |
| hemD |  |  |  |  |  |  |
| yihA |  |  |  |  |  |  |
| coaE |  |  |  |  |  |  |
| dnaN |  |  |  |  |  |  |
| ppa |  |  |  |  |  |  |
| guaA |  |  |  |  |  |  |
| lnt |  |  |  |  |  |  |
| ftsY |  |  |  |  |  |  |
| mreD |  |  |  |  |  |  |
| ispB |  |  |  |  |  |  |
| racR |  |  |  |  |  |  |
| glyQ |  |  |  |  |  |  |
| cydD |  |  |  |  |  |  |
| rpsA |  |  |  |  |  |  |
| higA |  |  |  |  |  |  |
| suhB |  |  |  |  |  |  |
| ispU |  |  |  |  |  |  |
| ubiD |  |  |  |  |  |  |
| nusA |  |  |  |  |  |  |
| secD |  |  |  |  |  |  |
| lolD |  |  |  |  |  |  |
| erpA |  |  |  |  |  |  |
| cydC |  |  |  |  |  |  |
| folE |  |  |  |  |  |  |
| 311 |  |  |  |  |  |  |

**Supplementary Table 3**

**Comparison of sequence reads mapping to *Ins* genes using different methods**

| **Sample Label** | **Total reads** | **Number of reads mapping >=1 time** | **Number of reads mapping once** |
| --- | --- | --- | --- |
| LoRTIS replicate 1 | 4,206,713 | 19,053 | 8,550 |
| LoRTIS replicate 1 | 7,629,034 | 28,172 | 13,812 |
| Illumina reads replicate 1 (Randomly selected comparable to read numbers of LoRTIS replicate 1) | 4,206,713 | 10,003 | 2,331 |
| Illumina reads replicate 2 (Randomly selected comparable to read numbers LoRTIS replicate 2) | 7,629,034 | 18,349 | 4,280 |

**Supplementary Table 4. Comparison between Illumina and Nanopore costs**

|  | **Illumina** | **Nanopore** |
| --- | --- | --- |
| **Instrument price** | £80.000-£500,000 | £500 |
| **Laboratory time (for generation of sequences)** | 20–56 h | 48 h |
| **Input DNA required** | 1–50 ng | 100–1000 ng |
| **Library preparation** | ~£50 | ~£120 |
| **Likely error rate** | Low | High |
| **Long reads** | No | Yes |
| **Requirement for diversity of sequencing library** | Yes | No |
| **Potential for single sample run** | No (challenging) | Yes |

**Supplementary Table 5. Primers used for LoRTIS**

| **Label** | **Primer sequence** |
| --- | --- |
| BioTn5Km-21 | GAGCTGTTGACAATTAATCATCGGCTCG |
| BioTn5Km-23 | GCTGAGTTGAAGGATCAGATCACGCATCTTC |
| IonTMu-02 | CGCGTTTTTCGTGCGTCAGTTCA |
| Nano4001 | GGTGCTGAAGAAAGTTGTCGGTGTCTTTGTGTTAACCTCGCGTTTTTCGTGCGTCAGTTCA |
| Nano4004 | GGTGCTGTTCGGATTCTATCGTGTTTCCCTATTAACCTCGCGTTTTTCGTGCGTCAGTTCA |
| Nano4005 | GGTGCTGCTTGTCCAGGGTTTGTGTAACCTTTTAACCTCGCGTTTTTCGTGCGTCAGTTCA |
| Nano4008 | GGTGCTGTTCAGGGAACAAACCAAGTTACGTTTAACCTCGCGTTTTTCGTGCGTCAGTTCA |
| Nano-5001 | GGTGCTGAAGAAAGTTGTCGGTGTCTTTGTGTTAACCTGATAACAATTTCACACAGGAAACAGCC |
| Nano-5004 | GGTGCTGTTCGGATTCTATCGTGTTTCCCTATTAACCTGATAACAATTTCACACAGGAAACAGCC |
| Nano-5005 | GGTGCTGCTTGTCCAGGGTTTGTGTAACCTTTTAACCTGATAACAATTTCACACAGGAAACAGCC |
| Nano-5008 | GGTGCTGTTCAGGGAACAAACCAAGTTACGTTTAACCTGATAACAATTTCACACAGGAAACAGCC |

**Supplementary Table 6.** PCR1 for amplification of transposons containing fragments with Biotin tags

**PCR1a**

**Reaction mix**

LongAmp Taq DNA polymerase 2 µl

LongAmp Taq Reaction Buffer 10 µl

100 mM dNTPs 0.2 µl

Biotinylated primer (100µM) 0.2 µl

DNA template 400 ng

Water upto 50 µl

**PCR conditions**

Denaturation 94 ͦC 60 Seconds

Denaturation 94 ͦC 30 Seconds

Annealing 60 ͦC 60 Seconds X 20 cycles

Extension 65 ͦC 10 Minutes

Extension 65 ͦC 10 Minutes

**PCR1b**

**Reaction mix**

Reaction mix of PCR1a

100 mM dNTPs 0.2 µl

IonTMu-02 primer (100µM) 0.2 µl

**PCR conditions**

Denaturation 94 ͦC 60 Seconds

Denaturation 94 ͦC 30 Seconds

Annealing 60 ͦC 60 Seconds X 18 cycles

Extension 65 ͦC 10 Minutes

Extension 65 ͦC 10 Minutes

**PCR 2,** Nested PCR for amplification of transposon containing fragments and addition of adapters for multiplexing

**Reaction mix**

LongAmp Taq DNA polymerase 2 µl

LongAmp Taq Reaction Buffer 10 µl

100 mM dNTPs 0.2 µl

Transposon specific indexed primer (100µM)* 0.2 µl

Museek adapter specific indexed primer (100µM)* 0.2 µl

DNA template 20 µl

Water up to 50 µl

* The primers in Supplementary Table 5 labelled as Nano4001- Nano4008 are museek adapter specific primers and Nano5001- Nano5008 are transposon-specific primers. Each primer has a unique 24 bp sequence that makes it unique compared with the others and we can identify the DNA fragments accordingly as they contain these unique sequences.

**PCR conditions**

Denaturation 94 ͦC 60 Seconds

Denaturation 94 ͦC 30 Seconds

Annealing 60 ͦC 60 Seconds X 12 cycles

Extension 65 ͦC 10 Minutes

Extension 65 ͦC 10 Minutes
